# Relationship between Carotid Intima Media Thickness and Helminth Infections on Flores Island, Indonesia

**DOI:** 10.1371/journal.pone.0054855

**Published:** 2013-01-24

**Authors:** Aprilianto Eddy Wiria, Linda J. Wammes, Firdaus Hamid, Olaf M. Dekkers, Margaretta A. Prasetyani, Linda May, Maria M. M. Kaisar, Jaco J. Verweij, Jouke T. Tamsma, Felix Partono, Erliyani Sartono, Taniawati Supali, Maria Yazdanbakhsh, Johannes W. A. Smit

**Affiliations:** 1 Department of Parasitology, Faculty of Medicine, University of Indonesia, Jakarta, Indonesia; 2 Department of Parasitology, Leiden University Medical Center, Leiden, The Netherlands; 3 Department of Microbiology, Faculty of Medicine, Hasanuddin University, Makassar, Indonesia; 4 Department of Clinical Epidemiology, Leiden University Medical Center, Leiden, The Netherlands; 5 Vascular Medicine, Department of Endocrinology and General Internal Medicine, Leiden University Medical Center, Leiden, The Netherlands; 6 Department of Endocrinology and General Internal Medicine, Leiden University Medical Center, Leiden, The Netherlands; 7 Department of General Internal Medicine, Radboud University Medical Center, Nijmegen, The Netherlands; The George Washington University Medical Center, United States of America

## Abstract

**Objective:**

To examine the association between helminth infections and atherosclerosis.

**Background:**

Chronic helminth infection, which can lead to poor nutritional status and anti-inflammatory response, might protect against the development of atherosclerosis.

**Methods:**

A cross-sectional study was performed in Flores, Indonesia, an area highly endemic for soil-transmitted helminths (STH). Stool samples from 675 participants aged 18–80 years were collected and screened for *Trichuris trichiura* by microscopy and for *Ascaris lumbricoides*, *Necator americanus, Ancylostoma duodenale, and Strongyloides stercoralis* by qPCR. We collected data on body mass index (BMI), waist to hip ratio (WHR), blood pressure, fasting blood glucose (FBG), lipid, high sensitive C-reactive protein (hs-CRP), total immunoglobulin-E (TIgE) and *Escherichia coli* lipopolysaccharide stimulated cytokines (tumor necrosis factor and interleukin-10). In a subset of 301 elderly adults (≥40 years of age) carotid intima media thickness (cIMT) was measured.

**Results:**

Participants with any STH infection had lower BMI (kg/m2) (mean difference −0.66, 95%CI [−1.26, −0.06]), WHR (−0.01, [−0.02, −0.00]), total cholesterol (mmol/L) (−0.22, [−0.43, −0.01]) and LDL-cholesterol (mmol/L) (−0.20, [−0.39, −0.00]) than uninfected participants. After additional adjustment for BMI the association between helminth infection and total cholesterol (mean difference −0.17, 95%CI [−0.37, 0.03]) as well as LDL-cholesterol (−0.15, [−0.33, 0.04]) was less pronounced. BMI, WHR, and total cholesterol were negatively associated with number species of helminth co-infections. Participants with high TIgE, an indicator of exposure to helminths, had lower FBG, TC, and HDL. The association between TIgE and TC and HDL remained significant after adjustment with BMI. No clear association was found between STH infection or TIgE and mean cIMT.

**Conclusions:**

This cross-sectional study presents evidence that helminth infections were negatively associated with risk factors for cardiovascular disease, an association at least partially mediated by an effect on BMI. The significance of this finding needs to be determined.

## Introduction

Mortality from cardiovascular diseases (CVD) accounts for 30% of total global deaths [1]. CVD is no longer a disease of Western countries exclusively since 80% of all CVD deaths worldwide take place in developing countries. In many Asian countries, rapid socioeconomic development has led to a shift in infrastructure, technology and food supply that promotes over nutrition and sedentary lifestyle [2], [3]. The relationship between a disturbed energy balance resulting from decreased physical activity or excess consumption of high-energy foods and CVD has long been acknowledged, but there is now abundant evidence that inflammation plays a role in chronic non-communicable diseases, including CVD. Indeed, in CVD elevated levels of several inflammation-related markers such as interleukin 6 (IL-6), IL-8, tumor necrosis factor (TNF), and C-reactive protein (CRP) [4] have been reported. One particular modifier of the pathogenesis of CVD in non-western societies may be related to differences in infectious pressure between rural and urban societies.

Although helminth infections, vary in their lifecycle and clinical impact in humans, they appear to share the ability to decrease inflammation and subsequently the development of inflammatory diseases which may include CVD [5]. We and others have shown that various helminth infections, which are endemic in many non-western societies, induce regulatory T cells (Treg) in order to ensure their survival within an immune competent host [6]–[9]. Helminths, such as schistosomes, that establish a systemic infection or soil-transmitted helminths (STH), which are restricted to the intestine, are also known to reduce energy intake and to be associated with poor nutritional status [10], [11]. Interestingly, in a study with apoE−/− mice, the development of atherosclerotic lesions was reduced by approximately 50% in animals with *S. mansoni* infections [12] whereas lipid-lowering effects were mediated by factors released from *S. mansoni* eggs [13]. Moreover, in an animal model of helminth infection with *Nippostrongylus brasiliensis*, a gastrointestinal nematode, with a lifecycle similar to hookworm in humans, it has been shown that infection is associated with beneficial effects of reducing traditional CVD risk factors such as obesity and serum lipid levels [14]. So far, to our knowledge, no studies have been published on the association between STH infections and atherosclerosis in humans.

Carotid intima media thickness (cIMT), is a marker for subclinical atherosclerosis [15] and is strongly associated with risk of CVD [16]. Assessment of cIMT is widely used in large-scale observational and experimental research. We set out to study the relationship between helminth infections CVD risk factors and cIMT as marker for atherosclerosis in an area endemic for STH on Flores Island, Indonesia.

## Materials and Methods

### Study Objectives

The primary objective of the study was to investigate the association between helminth infections and cIMT as marker for subclinical atherosclerosis. Our hypothesis was that, since helminths might protect against CVD, cIMT is lower in subjects with helminth infections than in subjects without helminth infections. The secondary objective was to study the association between helminth infections as well as total immunoglobulin E (TIgE), an indicator of exposure to helminths [17], and conventional CVD risk factors, including body mass index (BMI), waist hip ratio (WHR), blood pressure (BP), fasting blood glucose (FBG), serum lipid profile and serum markers of inflammation.

### Study Population

The study area is the semi urban area of Nangapanda on Flores Island in Indonesia [18], [19]. The area of Nangapanda is endemic for STH infections but not for filarial nematodes [9]. In this area, a large project is being conducted on the relationship between helminth infections and the immune system (ImmunoSPIN study [18], [19]). For the current study, a cross sectional representative sample was included from all inhabitants aged 18 years and above. Data were collected between May and August 2009.

### Study Design

From 2799 inhabitants from the Nangapanda area who participated in the ImmunoSPIN project, 691 were randomly selected to participate in the present cross-sectional study and invited to provide data on BMI, WHR, BP, and blood sampling for fasting glucose measurements, lipid profiles, TIgE, hs-CRP and whole blood culture to stimulate cytokine production. Data on helminth infections, BP, BMI and WHR ratio were available from 675 subjects included in the present analysis. In 595 of these subjects, laboratory measurements were performed. Carotid artery IMT was measured in a subset of 301 adult participants above 40 years of age.

The study was approved by The Ethical Committee of Faculty of Medicine, University of Indonesia, ref: 194/PT02.FK/Etik/2006 with addendum ref: 96/PT02.FK/Etik/2010 and registered as clinical trial ref: ISRCTN83830814 and was filed by the Leiden University Medical Center Committee of Medical Ethics (CME). Because of the high rate of illiteracy amongst elderly participants, either written or verbal informed consent was obtained from each participant.

### Clinical and Laboratory Assessment

Anthropometric measurement of body weight (SECA 761, SECA GMBH & Co. Kg., Hamburg, Germany), height (SECA 206, SECA GMBH & Co. Kg., Hamburg, Germany), waist and hip circumference (WC and HC) (SECA 203, SECA GMBH & Co. Kg., Hamburg, Germany) were obtained using the NHLBI practical guidelines (NHLBI web). Three blood pressure (BP) measurements (left arm, sitting upright position, after resting 5 minutes) were taken from each subject, using a digital Omron sphygmomanometer (705IT HEM-759P-E2, OMRON Healthcare Europe BV, The Netherlands), and calibrated using a Riester nova-presameter®-Desk model mercury sphygmomanometer (Gerhard Glufke Rudolf Riester GmbH & Co, Jungingen, Germany) and a 3M™ Littmann® Classic II S.E. Stethoscope (3M, St. Paul, Minnesota, USA). The average of three systolic/diastolic BP measurements was used. Abnormal BMI is ≥25 kg/m^2^ and the Asian modified abnormal waist hip ratio (WHR) is >0.9 (men) and >0.8 (women) [20]. Abnormal blood pressure was considered as hypertension when BP≥140/90 mmHg.

All participants were instructed to be fasting before venous sampling. FBG was analyzed using Breeze®2 glucose meter (Bayer Health Care LLC, Basel, Switzerland). Lipid profile was measured using commercial enzymatic kits for total cholesterol (TC), high density lipoprotein-cholesterol (HDL-c) and triglycerides (TG) (Roche Molecular Biochemicals, Indianapolis, USA) and determined using ELISA reader (LabSystem Multiscan, MHC347, Helsinski, Finland). Low density lipoprotein-cholesterol (LDL-c) was calculated by using the Friedwald calculation [21]. High sensitive C-reactive protein (hs-CRP) level was measured using MSD® 96-Well MULTI-ARRAY® CRP Assay (Meso Scale Discovery, Gaithersburg, USA). TIgE level was measured by an ELISA as described in detail previously [18], [19].

### Helminth Status

Stool samples were collected and preserved in 4% formaldehyde for microscopy examination or frozen (−20°C) unpreserved for PCR detection. The formol-ether acetate concentration method was performed on the formalin preserved stool samples followed by microscopy examination for *Trichuris trichiura* infections [19]. As described in detail before [19] the DNA of *Ancylostoma duodenale*, *Necator americanus*, *Ascaris lumbricoides* and *Strongyloides stercoralis* were isolated from approximately 100 mg unpreserved faeces and were examined by the multiplex qPCR. The qPCR output from this system consisted of a cycle-threshold (CT) value, representing the amplification cycle in which the level of fluorescent signal exceeds the background fluorescence, and reflecting the parasite-specific DNA load in the sample tested. Negative and positive control samples were included in each run of the amplification. We defined a positive case for *T. trichiura* by the egg findings and for *A. duodenale*, *N. americanus*, *A. lumbricoides* and *S. stercoralis* by parasite-specific DNA findings. Participants were also grouped by number of helminth co-infections.

### Carotid Intima Media Thickness

We used ultrasound for measuring cIMT [22]. Quality control and details of the IMT measurement have been described before [23]. cIMT was measured while the participant was lying in a supine position. Measurements were made at 3 different angles of both the right and left common carotid artery at 10 mm proximal of the carotid artery bulb using a mobile device: Mylab®25 ultrasound system with a LA523 13-4 MHz transducer (ESAOTE, S.p.A, Maastricht, The Netherlands). The mean of these 6 measurements was used in the analysis. In order to keep variation minimal, one of the physicians (AEW) performed all intima-media thicknesses measurements on the participants in this study.

### Whole Blood Stimulation and Cytokine Measurement

The procedure of whole blood stimulation and cytokines measurement has been described previously [19]. Briefly, heparinized blood within 6 hours of blood draws was diluted 4× and stimulated. Stimulations were performed with control medium or *Escherichia coli* lipopolysaccharide (LPS, 1 ng/ml Sigma-Aldrich, Zwijndrecht, The Netherlands), incubated for 24 hours at 37°C and 5% CO_2_. The supernatants were frozen at −20°C and transported to Jakarta where TNF and IL-10 were assessed by means of immunobead-based multiplex assays (Biosource, Camarillo, CA, USA) on a Liquichip 200® Workstation (Qiagen, Venlo, The Netherlands) using Liquichip analyzer software (Qiagen, Venlo, The Netherlands). Samples with TNF levels higher than 250 pg/ml in medium stimulation were excluded from further analyses (2 samples).

### Statistical Analysis

Participant characteristics were stratified for helminth infection (uninfected and infected). Linear regression was used to study the associations between helminth infections and also number of helminth species co-infection and risk factors for CVD adjusted for age and sex. Differences between infected and uninfected participants were reported as mean differences with 95% confidence intervals (95% CI). TIgE, cytokines and CRP concentrations were normalized by log-transformation, analyses were performed with these log transformed values but results were presented as geometric means after exponentiation of the values on a logarithmic scale. To assess whether a potential association between helminth infection and CVD risk factors is mediated through an effect on BMI, we performed a second analysis additionally adjusted for BMI. Similar analyses were performed on the cIMT subset participants. Furthermore, in the helminth infected group we tested the association of helminth load per species with CVD risk factors. In addition, as high TIgE is associated with exposure to helminth infections, we tested the association between TIgE and risk factors for CVD as well as cIMT. Subjects were considered to have high or low TIgE based on the value above or below and equal the geometric mean. P values <0.05 were considered to be statistically significant. Statistical analysis was done using SPSS statistics 17.0.2 (SPSS Inc., Chicago, Illinois, USA).

## Results

### Characteristics of Study Participants

A total of 446 participants infected with at least one helminth were compared to 229 uninfected participants. There were more males in the infected group (37.7%) than in the uninfected group (34.1%), whereas the mean age was similar (45.0 vs. 44.8 years). The most prevalent STH infections were *N. americanus* 348/675 (51.6%), *A. lumbricoides* 149/675 (22.1%) and *T. trichiura* 139/675 (20.6%). The proportion of participants infected with *A. duodenale* 24/675 (3.6%) and with *S. stercoralis* 5/675 (0.7%) was clearly lower. 273 participants were infected with one helminth only, 131 with two helminths and 42 with 3 or more helminths species. As expected, TIgE was higher in individuals infected with STH infection. (Table 1).

**Table 1:** Characteristics of the study population regarding helminth uninfected and infected.

	Helminth uninfected(n = 229)	Helminth infected(n = 446)	Mean difference adjusted for age and sex(95% confidence interval)	Mean difference adjusted for age, sex and BMI(95% confidence interval)
**Age (year) (mean, Range)**	44.8 (18.2–80.2)	45.0 (18.0–79.5)	–	–
**Male (%)**	34.1	37.7	–	–
***Trichuris trichiura^1^*** ** (%)**	0	31.2	–	–
***Ascaris lumbricoides^2^*** ** (%)**	0	33.4	–	–
***Necator americanus^2^*** ** (%)**	0	78.0	–	–
***Ancylostoma duodenale^2^ (%)***	0	5.4	–	–
***Strongyloides stercoralis^2^ (%)***	0	1.1	–	–
**BMI (Kg/m2) (mean, SD)**	23.1 (3.7)	22.5 (3.8)	−0.66 (−1.26, −0.06), p = 0.031	–
**WHR (mean, SD)**	0.89 (0.07)	0.88 (0.06)	−0.01 (−0.02, −0.00), p = 0.011	–
**Systole (mmHg) (mean, SD)**	130.9 (22.5)	129.8 (24.4)	−1.18 (−4.56, 2.19), p = 0.49	−0.38 (−3.71, 2.95), p = 0.82
**Diastole (mmHg) (mean, SD)**	78.5 (12.2)	76.7 (12.6)	−1.76 (−3.71, 0.20), p = 0.078	−1.28 (−3.17, 0.61), p = 0.19
**FBG (mmol/L) (mean, SD)**	5.9 (1.5)	5.9 (1.6)	−0.05 (−0.31, 0.21), p = 0.71	0.02 (−0.24, 0.27), p = 0.89
**TC (mmol/L) (mean, SD)**	5.1 (1.2)	4.9 (1.1)	−0.22 (−0.43, −0.01), p = 0.037	−0.17 (−0.37, 0.03), p = 0.098
**HDL-c (mmol/L) (mean, SD)**	1.6 (0.4)	1.5 (0.4)	−0.02 (−0.09, 0.05), p = 0.49	−0.03 (−0.10, 0.04), p = 0.34
**TG (mmol/L) (mean, SD)**	1.2 (0.7)	1.3 (0.7)	0.00 (−0.13, 0.13), p = 1.00	0.05 (−0.07, 0.17), p = 0.45
**LDL-c (mmol/L) (mean, SD)**	3.3 (1.1)	3.1 (1.0)	−0.20 (−0.39, −0.00), p = 0.048	−0.15 (−0.33, 0.04), p = 0.13
**TC to HDL-c ratio (mean, SD)**	3.5 (1.1)	3.3 (1.0)	−0.09 (−0.27, 0.10), p = 0.38	−0.03 (−0.21, 0.15), p = 0.74
**TIgE (IU/ml) (geometric mean** [95%CI])*	834.3 (716.5–971.5)	1182.1 (1044.5–1337.8)	1.42 (1.15, 1.75), p = 0.0010	1.40 (1.14, 1.73), p = 0.0016
**hs-CRP (mg/l) (geometric mean** [95%CI])*	0.5 (0.4–0.6)	0.5 (0.4–0.6)	1.15 (0.74, 1.30), p = 0.90	1.16 (0.77, 1.37), p = 0.85
**TNF (pg/ml) (geometric mean** [95%CI])*	217.8 (175.3–270.5)	278.6 (243.7–318.4)	1.28 (1.00, 1.64), p = 0.050	1.27 (0.99, 1.64), p = 0.062
**IL-10 (pg/ml) (geometric mean** [95%CI])*	128.0 (105.4–155.6)	125.5 (111.7–140.0)	0.98 (0.79, 1.22), p = 0.87	0.98 (0.99, 1.22), p = 0.85

1. positive by microscopy.

2. positive by PCR.

Abbreviations: BMI = body mass index, WHR = waist to hips ratio, FBG = fasting blood glucose, TC = total cholesterol, TG = triglyceride, HDL-c = high density lipoprotein cholesterol, LDL-c = low density lipoprotein cholesterol, TIgE = total immunoglobulin E, hs-CRP = high sensitive C reactive protein, TNF = tumor necrosis factor, IL-10 = interleukin 10 cytokines were stimulated for 24 h with *E. coli* lipopolysaccharide (LPS). *Adjusted mean difference for TIgE, hs-CRP and cytokines were anti-log transformed and represent ratio.

### Association between Helminth Infection and CVD Risk Factors

Participants with any helminth infection had lower BMI (mean difference −0.66, 95%CI −1.26, −0.06), WHR (−0.01, 95%CI −0.02, −0.00), total cholesterol (−0.22, 95%CI −0.43, −0.01) and LDL-cholesterol (−0.20, 95% CI −0.39, −0.00) than uninfected participants (Table 1). After additional adjustment for BMI the association between helminth infection and total cholesterol (mean difference −0.17, 95%CI −0.37, 0.03) as well as LDL-cholesterol (−0.15, 95%CI −0.33, 0.04) was less pronounced. No clear associations were found between helminth infection and blood pressure, fasting blood glucose and triglycerides. No difference in hs-CRP between helminth-infected and –uninfected groups was found.

Next, we analyzed whether the number of helminth species infecting a participant was related to CVD risk factors (data not shown). BMI, WHR, and total cholesterol were negatively associated with the number of helminth infections. Adjustment for BMI attenuated the association between number of infections and total cholesterol. No marked associations were found between number of infections and blood pressure, LDL, HDL, triglycerides or fasting glucose levels. In addition, TNF production in response to LPS stimulation was positively associated with the number of helminth infections with the highest LPS-TNF levels in participants with 3 or more infections. This association remained after adjustment for BMI (p = 0.03).

Looking at intensity of infection; in infected participants no associations were found between *N. americanus* load (as measured by qPCR) and BMI, WHR, fasting blood glucose, blood pressure or cholesterol levels. A negative association was found between *A. lumbricoides* load and triglyceride independent of BMI (P<0.001).

### The Association between Helminth Infections and IMT

IMT was measured in 70% of participants ≥40 years (table 2). In accordance with the whole study population, BMI, WHR, TC and LDL were lower in helminth infected patients. IMT was only marginally lower in infected participants than in uninfected participants (mean difference −4.7, 95% CI −27.7, 18.3). In addition, no relationships between the *number* and load of helminth infections and IMT were found. No differences in IMT were found between *N. americanus* infected participants and non-infected participants either.

**Table 2:** Characteristic of the group with intima media thickness measurement (population 40+).

	Helminthuninfected(n = 140)	Helminthinfected(n = 291)	Mean difference adjusted for age and sex(95% confidence interval)	Mean difference adjusted for age, sex and BMI(95% confidence interval)
**Age (year) (mean, Range)**	53.4 (40.0–80.2)	53.6 (40.5–79.5)	–	–
**Male (%)**	40.7	45.7	–	–
***Trichuris trichiura^1^*** ** (%)**	0	31.6	–	–
***Ascaris lumbricoides^2^*** ** (%)**	0	28.9	–	–
***Necator americanus^2^*** ** (%)**	0	79.4	–	–
***Ancylostoma duodenale^2^ (%)***	0	5.5	–	–
***Strongyloides stercoralis^2^ (%)***	0	1.4	–	–
**IMT (≥40 years old) (µm) (mean, SD)**	654 (122)	650 (112)	−4.68 (−27.65, 18.29), p = 0.69	−0.95 (−24.17, 22.27), p = 0.94
**BMI (Kg/m2) (mean, SD)**	23.2 (3.7)	22.2 (3.8)	−0.91 (−1.65, −0.17), p = 0.017	–
**WHR (mean, SD)**	0.89 (0.07)	0.88 (0.06)	−0.02 (−0.03, −0.00), p = 0.015	–
**Systole (mmHg) (mean, SD)**	139.8 (23.4)	136.8 (26.1)	−3.08 (−8.06, 1.91), p = 0.23	−1.52 (−6.43, 3.39), p = 0.54
**Diastole (mmHg) (mean, SD)**	81.3 (13.7)	78.2 (13.4)	−2.99 (−5.75, −0.24), p = 0.033	−2.12 (−4.77, 0.53), p = 0.12
**FBG (mmol/L) (mean, SD)**	6.1 (1.8)	6.1 (1.9)	−0.01 (−0.41, 0.39), p = 0.96	0.10 (−0.29, 0.49), p = 0.62
**TC (mmol/L) (mean, SD)**	5.2 (1.3)	5.0 (1.1)	−0.24 (−0.50, −0.01), p = 0.063	−0.18 (−0.43, 0.08), p = 0.17
**HDL-c (mmol/L) (mean, SD)**	1.5 (0.4)	1.5 (0.4)	−0.03 (−0.12, 0.06), p = 0.49	−0.05 (−0.13, 0.04), p = 0.31
**TG (mmol/L) (mean, SD)**	1.4 (0.6)	1.4 (0.8)	−0.01 (−0.18, 0.16), p = 0.89	0.07 (−0.10, 0.23), p = 0.43
**LDL-c (mmol/L) (mean, SD)**	3.4 (1.1)	3.2 (1.0)	−0.21 (−0.45, 0.03), p = 0.085	−0.15 (−0.38, 0.09), p = 0.23
**TC to HDL-c ratio (mean, SD)**	3.5 (1.1)	3.3 (1.0)	−0.10 (−0.34, 0.14), p = 0.40	−0.03 (−0.26, 0.21), p = 0.82
**TIgE (IU/ml) (geometric mean** [95%CI])*	743.3 (613.5–900.5)	1080.3 (927.5–1258.3)	1.41 (1.09, 1.84), p = 0.010	1.39 (1.07, 1.82), p = 0.015
**hs-CRP (mg/l) (geometric mean** [95%CI])*	0.6 [0.4–0.7]	0.6 [0.5–0.7]	1.20 (0.77, 1.37), p = 0.88	1.21 (0.74, 1.55), p = 0.72
**TNF (pg/ml) (geometric mean** [95%CI])*	223.5 [168.2–297.0]	265.4 [226.5–310.6]	1.21 (0.89, 1.65), p = 0.22	1.18 (0.85, 1.62), p = 0.32
**IL-10 (pg/ml) (geometric mean** [95%CI])*	123.9 [95.3–161.1]	119.2 [104.3–136.2]	0.97 (0.74, 1.27), p = 0.81	0.96 (0.73, 1.27), p = 0.77

1. positive by microscopy.

2. positive by PCR.

Abbreviations: BMI = body mass index, WHR = waist to hips ratio, FBG = fasting blood glucose, TC = total cholesterol, TG = triglyceride, HDL-c = high density lipoprotein cholesterol, LDL-c = low density lipoprotein cholesterol, TIgE = total immunoglobulin E, hs-CRP = high sensitive C reactive protein, TNF = tumor necrosis factor, IL-10 = interleukin 10. *Adjusted mean difference for TIgE, hs-CRP and cytokines were anti-log transformed and represent ratio.

### The Association between TIgE Level, CVD risk Factors and cIMT

A negative association was found between TIgE and FBG (mean difference −0.31, 95%CI −0.61, −0.02), TC (−0.23, 95%CI −0.42, −0.03), and HDL (−0.08, 95%CI −0.14, −0.01). The association remained significant after adjustment for BMI for TNF (1.22, 95%CI 0.96, 1.57) and IL-10 (1.27, 95%CI 1.03, 1.57). No difference was found between TIgE and cIMT. In analyses using continuous levels of TIgE, we found a significant association between TIgE and TNF.

## Discussion

The objective of this study was to examine the association between helminth infections and atherosclerosis in a population residing in an area highly endemic for STH. The hypothesis being tested is that helminth infections may have a beneficial effect on the development of atherosclerosis, both by influencing conventional CVD risk factors and systemic inflammation. Although this beneficial effect has been illustrated in animal studies [12], [13], no human studies on the relationship between STH infections and atherosclerosis have been published before.

We found a negative association between helminth infections and conventional CVD risk factors, including BMI, WHR and serum cholesterol levels, which was independent of age and gender. In addition, with increasing number of helminth species, the negative association with CVD risk factors increased. The association was similar when we analyzed TIgE as a marker of exposure to helminth infections and reflects not only current but also prior exposure to helminth infection [17].

The finding of lower lipid levels in individuals with infections in our study area is similar to what was found in Tsimane [24] or Shipibo [25] population. The lipids measured included HDL, which might seem surprising as decreased HDL level is considered to be a CVD risk factor. Interestingly, the anti-atherogenic properties of HDL might be dependent on the composition of HDL and the context in terms of type of inflammation [26]. It would be important to investigate whether helminth infections affect the type of HDL or its role in cholesterol transport.

With regards to cytokine production, we found a positive association of TNF production in response to LPS stimulation with helminth infections as well as with TIgE. These data suggest that helminths are associated with pro-inflammatory responses. Although higher TNF and pro-inflammatory cytokines have been found in the circulation of helminth infected patients with pathology [27]–[29], most studies of subjects infected with helminths with no overt pathology, report an anti-inflammatory effect of these parasites on the immune system. However, the before mentioned have focused on the adaptive responses and include Tregs [6]–[9]. It should be noted that, in line with the current data, a recent study in Gabon found that *S. haematobium* infected children develop a more pro-inflammatory TLR-mediated response [30]. We did not observe substantial differences in STH species and their effects on CVD risk factors.

Although we showed a relationship between helminth infections and traditional CVD risk factors, we did not find an association between helminth infections and cIMT. The following explanations may account for the absence of a relationship between helminth infection status and cIMT. In our study, helminth infection was assessed at one time-point. Theoretically, it may be possible that helminth-negative subjects in our study have only recently become helminth negative. In this situation, the beneficial effects of helminths can be seen on some of the traditional CVD risk factors, but not atherosclerosis development, as the dewormed state may have been too short to affect cIMT. As the development of atherosclerosis is a chronic process, ideally, lifetime exposure to helminth infections should be assessed in relation to atherosclerosis development. Although this is not feasible, the association was also not significant when TIgE, reflecting current and prior helminth infection, was considered. Another explanation is that the population studied has a low cardiovascular risk profile. Although the association of CVD risk factors and IMT in the Flores population was similar to other studies [4], [31]–[34], mean IMT in the study population was lower than comparable age groups in Europe [35], the USA [36], and Japan [37]. Indeed most other CVD risk parameters were well within the normal reference range. In this situation, the absolute levels of CVD risk factors may be below the threshold for accelerated atherosclerosis development.

In conclusion, in a large cross-sectional study in an area endemic for helminth infections, we found an association between STH infection and conventional risk factors for CVD. The effect of helminth infections on CVD was at least partially mediated by an effect on BMI. However, no direct association between helminth infections and IMT was found. Nevertheless we believe that this study shows that in the explanation of increase in CVD in non-western societies, changes in infectious pressure should be taken into account. Further studies are necessary to assess the causal relationships between helminth infections and atherosclerosis.
